# Effect of fasting and feeding on apolipoprotein A-I kinetics in preβ_1_-HDL, α-HDL, and triglyceride-rich lipoproteins

**DOI:** 10.1038/s41598-020-72323-w

**Published:** 2020-09-24

**Authors:** Maud Chétiveaux, Mikaël Croyal, Khadija Ouguerram, Fanta Fall, Laurent Flet, Yassine Zair, Estelle Nobecourt, Michel Krempf

**Affiliations:** 1 CRNH-O Mass Spectrometry Core Facility, Nantes, France; 2 NUN, INRA, CHU Nantes, UMR 1280, PhAN, IMAD, CRNH-O, IRS-UN–Spectrométrie de Masse-8, quai Moncousu, 44000 Nantes, France; 3 Pharmacy Department, Nantes University Hospital, Nantes, France; 4 Nephrology Department, CHU Saint-Pierre, La Réunion, France; 5 Clinique Bretéché, Groupe Elsan, Nantes, France

**Keywords:** Biochemistry, Physiology, Biomarkers

## Abstract

The aim of this study was to compare the kinetics of apolipoprotein (apo)A-I during fed and fasted states in humans, and to determine to what extent the intestine contributes to apoA-I production. A stable isotope study was conducted to determine the kinetics of apoA-I in preβ_1_ high-density lipoprotein (HDL) and α-HDL. Six healthy male subjects received a constant intravenous infusion of ^2^H_3_-leucine for 14 h. Subjects in the fed group also received small hourly meals. Blood samples were collected hourly during tracer infusion and then daily for 4 days. Tracer enrichments were measured by mass spectrometry and then fitted to a compartmental model using asymptotic plateau of very-low-density lipoprotein (VLDL) apoB100 and triglyceride-rich lipoprotein (TRL) apoB48 as estimates of hepatic and intestinal precursor pools, respectively. The clearance rate of preβ_1_-HDL-apoA-I was lower in fed individuals compared with fasted subjects (*p* < 0.05). No other differences in apoA-I production or clearance rates were observed between the groups. No significant correlation was observed between plasma apoC-III concentrations and apoA-I kinetic data. In contrast, HDL-apoC-III was inversely correlated with the conversion of α-HDL to preβ_1_-HDL. Total apoA-I synthesis was not significantly increased in fed subjects. Hepatic production was not significantly different between the fed group (17.17 ± 2.75 mg/kg/day) and the fasted group (18.67 ± 1.69 mg/kg/day). Increase in intestinal apoA-I secretion in fed subjects was 2.20 ± 0.61 mg/kg/day. The HDL-apoA-I kinetics were similar in the fasted and fed groups, with 13% of the total apoA-I originating from the intestine with feeding.

## Introduction

Epidemiological studies have suggested that high-density lipoproteins (HDL) could prevent coronary heart disease but genetic cohort studies or trials investigating HDL- targeted therapies did not give strong supports to this finding^1^. The metabolism and the physiological role of HDL is not fully understood and is not probably only resumed to the reverse transport of cholesterol from the macrophages to the liver^2^. Then, the function and metabolism of HDL need to be revisited. Apolipoprotein A-I (apoA-I) constitutes the major apolipoprotein component of the HDL and is a major driver of their metabolism^3^. ApoA-I is produced by both the liver and the intestine and its synthesis is affected by many factors including diet^2,3^. However, little is known about the intestinal production rate of apoA-I and the differential utilization of the HDL originating from these both sources.

Numerous kinetic studies with stable isotope tracers determined kinetic parameters of HDL-associated apoA-I. However, most of them were performed with subjects in either fasted or fed state, without direct comparisons between both study designs. Hence, the impact of fasted/fed status on HDL and apoA-I metabolism, especially within the intestine, remains not clearly understood. To address this issue, Cohn et al*.* analyzed HDL metabolism by exogenous labeling of apoA-I and they did not observe any change in HDL-apoA-I pool size, production rate, or catabolic rate in either the fasted or the fed state^3^. However, this study only analyzed mature HDL (assumed to be HDL_2_ and HDL_3_) which could not account for intestinal contributions.

HDL metabolism is a complex process involving precursor (preβ_1_-HDL) and mature (α-HDL) particles^2,4^. Kinetic studies discriminating preβ_1_-HDL and α-HDL subpopulations and with a direct comparison in fasted and fed subjects have never been performed. Such studies have not been undertaken likely due to the complexity of preβ_1_-HDL metabolism, which is recycled in other HDL populations and originates from two different sources: (1) a direct synthesis from the liver and the intestine^5^, and (2) an indirect synthesis from intestine-produced postprandial triglyceride-rich lipoproteins (TRL)^6^, which have undergone hydrolysis by lipoprotein lipase^7,8^.Of note, apolipoprotein C-III (apoC-III) inhibits TRL hydrolysis by acting on lipoprotein lipase^9^ and food intake increases apoC-III plasma concentrations^10,11^. The pharmacological inhibition of apoC-III production with an antisense oligonucleotide dramatically increased HDL cholesterol (HDL-C) by 40% in both healthy and hypertriglyceridemic subjects^12^.

We previously reported a kinetic study examining the production of preβ_1_-HDL during fasting state^13^. Here, we used the same approach to compare apoA-I kinetics in TRL, preβ_1_-HDL, and α-HDL subpopulations in healthy subjects during fasted and fed states. Our goals were to analyze the effects of food intake and therefore plasma apoC-III on HDL metabolism, and to estimate the intestinal production rate of apoA-I.

## Results

### Plasma lipids and apolipoprotein concentrations

**Figure 1:**
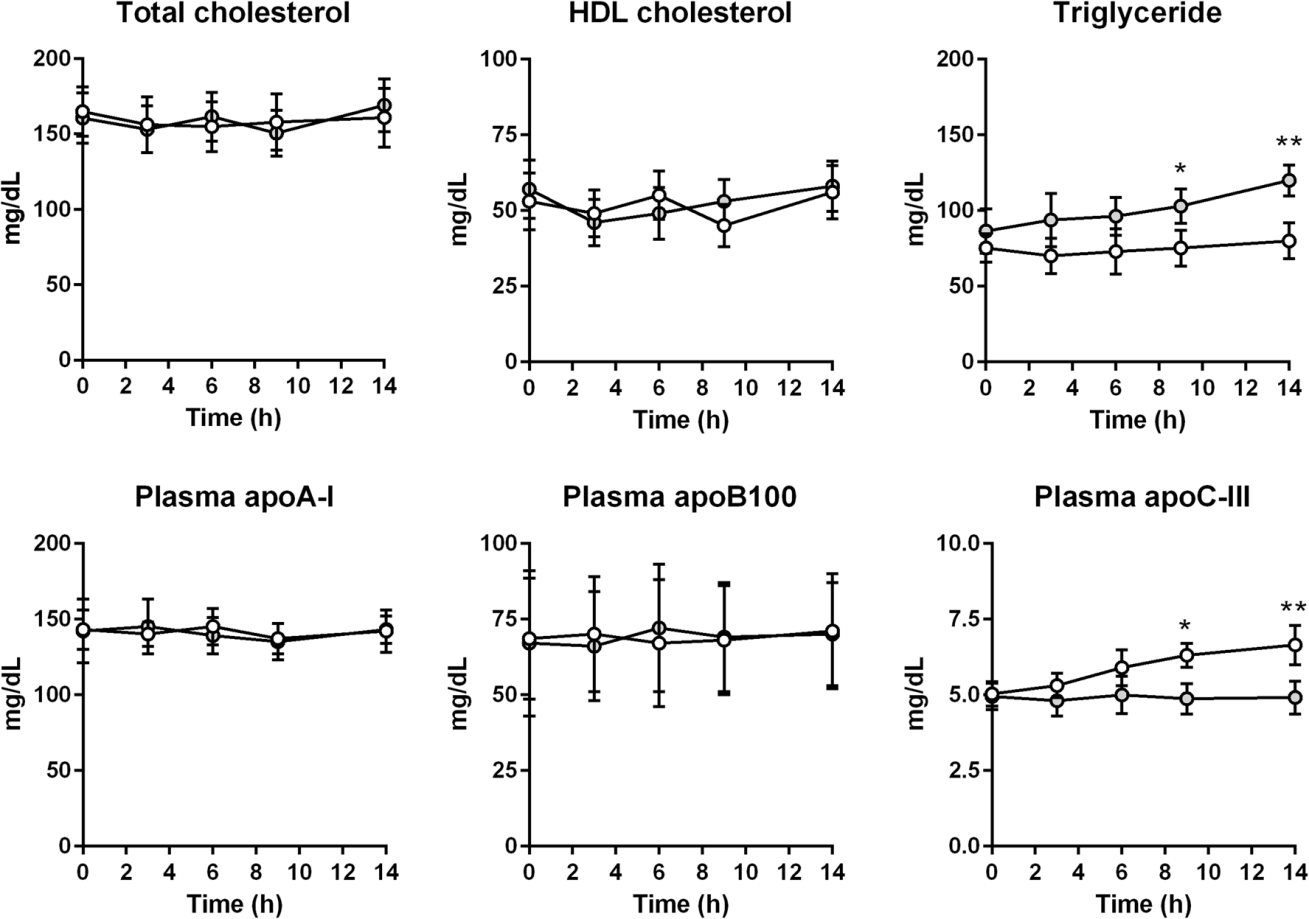
Evolution of main lipid parameters during the kinetic protocols. The white circles indicate the fasted sate and the grey circles indicate the feeding state. Values represent means ± SD for six subjects. *p < 0.05; **p < 0.01 (Wilcoxon matched-pairs signed rank test).

As shown in Fig. 1, plasma total cholesterol (TC) concentrations did not change during the tracer infusion protocol and were similar in both the fasted and fed states. In the fasted state, plasma triglyceride (TG) concentrations remained unchanged and stable. In the fed state plasma TG concentrations were similar at baseline compared to the fasted state, then shortly increased significantly (110 ± 15 mg/dL vs. 67 ± 7 mg/dL, *p* < 0.001). HDL cholesterol (HDL-C), apolipoprotein B100 (apoB100) and apoA-I concentrations were kept constant with no difference between both experiments. While fasting did not affect plasma apoC-III concentrations (4.4 ± 0.4 mg/L at 14 h vs. 5.0 ± 0.4 mg/dL at baseline), feeding significantly increased plasma apoC-III by 30 ± 7% at 14 h (*p* = 0.009). No significant difference was found for HDL-apoC-III between both the fasted and feeding states (2.9 ± 1.3 mg/dL vs. 2.7 ± 0.9 mg/dL).

### Plasma leucine enrichments

**Figure 2:**
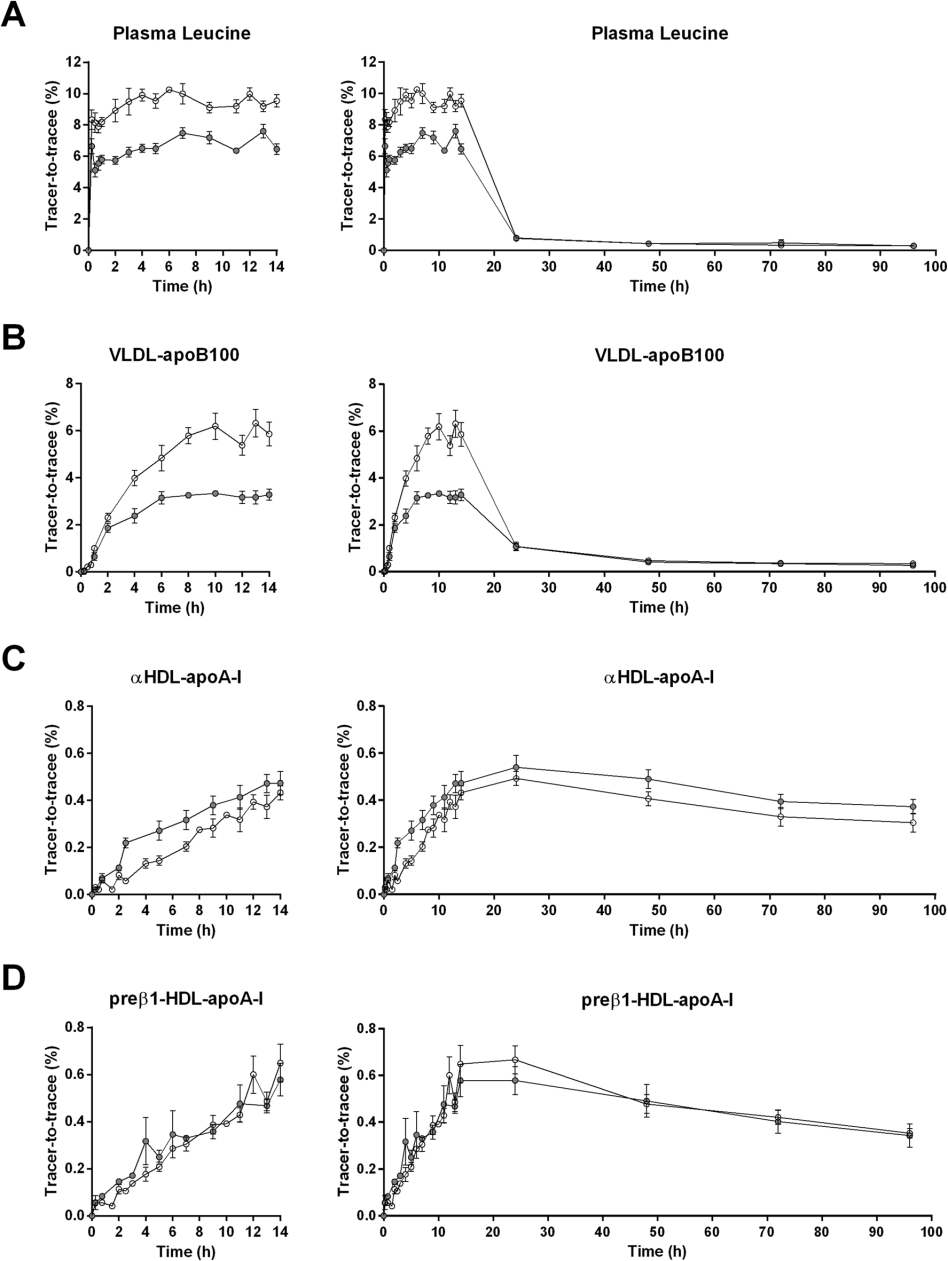
Enrichment of plasma leucine (**A**), VLDL-apoB100 (**B**), preβ_1_-HDL-apoA-I (**C**) and α-HDL-apoA-I (**D**) with deuterated leucine during the fasted (white circles) and fed state (grey circles) over 14 h or 96 h. Values represent means ± SD for six subjects.

Plasma leucine enrichments increased rapidly after initial tracer injection and remained relatively constant for the 14 h duration of the studies (Fig. 2). The mean plasma leucine enrichments were lower in the fed state compared with the fasted state at their respective plateaus (6.4 ± 0.7% vs. 9.2 ± 0.8%, *p* = 0.005). As expected, the flux rate of leucine was increased with feeding (99 ± 12 µmol/kg/h and 146 ± 15 µmol/kg/h for fasted and fed groups, respectively, *p* = 0.005).

### TRL-apoB48, VLDL-apoB100, HDL-apoA-I, and TRL-apoA-I enrichments

**Figure 3:**
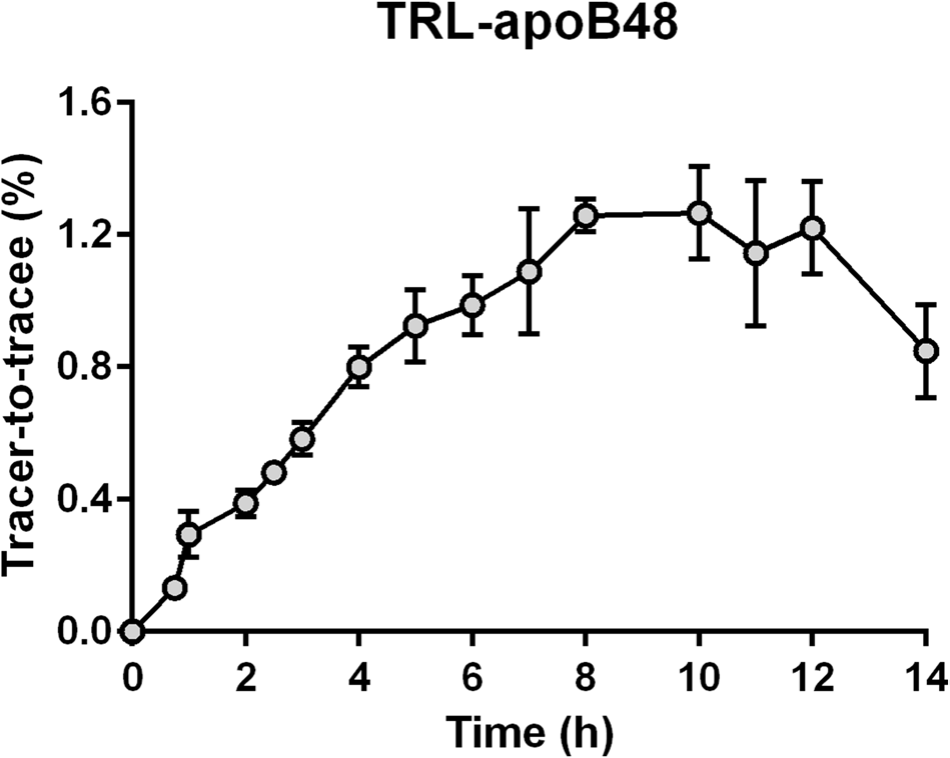
Enrichment of TRL-apoB48 with deuterated leucine during the fed state. Values represent means ± SD for six subjects.

**Figure 4:**
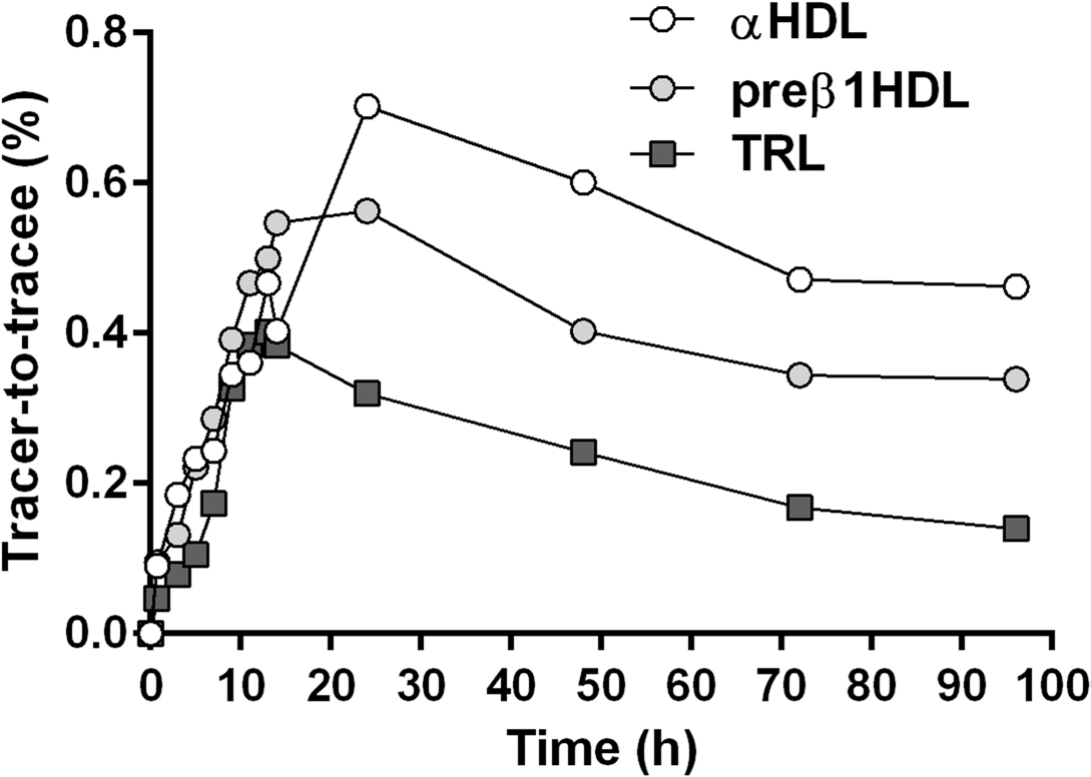
ApoA-I tracer/tracee ratios within TRL, preβ_1_-HDL and α-HDL fractions after a constant deuterated leucine infusion in a representative subject in a fed state.

A plateau in very-low-density lipoprotein (VLDL)-apoB100 ^2^H_3_-leucine enrichments was observed in both fasted and fed groups around 8 h after the start of the tracer infusion (Fig. 2). The plateau tracer enrichment of VLDL-apoB100 was significantly lower in the fed group relative to the fasted group (3.2 ± 0.9% vs. 6.5 ± 1.5%, respectively, *p* = 0.038) and TRL-apolipoprotein B48 (apoB48) was significantly lower than that of VLDL-apoB100 (1.4 ± 0.5% vs. 3.2 ± 0.9%, *p* = 0.005) (Fig. 3). Enrichment curves of α-HDL-apoA-I did not reach a plateau and tended to be lower in the fasted group compared to the fed group, although this difference was not significant. Preβ_1_-HDL-apoA-I enrichments were not different between both experimental groups (Fig. 2). During the infusion of the tracer, the enrichment curves were found similar for apoA-I in TRL, preβ_1_-HDL and α-HDL (Fig. 4) suggesting they are quickly exchanged and equilibrated. However, the decay curve of apoA-I enrichment upon termination of infusion was faster in TRL (0.185 ± 0.055 Atom percent excess (APE) at 96 h) than in HDL particles (0.343 ± 0.050 APE and 0.373 ± 0.030 APE at 96 h for preβ_1_-HDL and α-HDL, respectively).

### Kinetic parameters within lipoprotein subclasses

In the fed group, TRL-apoA-I and TRL-apoB48 displayed similar absolute production rates (APR), while TRL-apoB48 fractional catabolic rates (FCR) were found higher than that of TRL-apoA-I (*p* = 0.045, Table 1). Exchange of apoA-I between HDL and TRL particles was identified with a net transfer of apoA-I from TRL to HDL (1.40 ± 0.48 mg/kg/day). VLDL-apoB100 kinetic parameters (catabolism, synthesis, pool size) tended to be higher in the fed group compared with the fasted group (not significant). Synthesis and clearance rates of apoA-I in the preβ_1_-HDL and α-HDL particles were not statistically different in both fasted and fed groups, except for preβ_1_-HDL-apoA-I FCR that were higher in the fasted group (Table 2, 0.447 ± 0.067 vs. 0.235 ± 0.038 pool/day, *p* = 0.045). At the feeding state, TRL- and α-HDL-apoA-I FCR were of 4.29 ± 1.09 and 0.338 ± 0.035 pool/day, respectively. The residence time (RT) of TRL-apoA-I was 0.23 days compared with 2.98 days for α-HDL-apoA-I. Hepatic apoA-I APR was estimated to be 18.67 ± 1.69 and 17.17 ± 2.75 mg/kg/day for fasted and fed groups, respectively (not significant). We could not identify any direct production of apoA-I from the intestine within the preβ_1_-HDL population. Intestinal apoA-I production could only be estimated from apoA-I secretion within the TRL isolated in the fed group, at a rate of 2.20 ± 0.61 mg/kg/day. Therefore, 13 ± 9% of the total body apoA-I production rate could be attributed to the intestine when feeding.

**Table 1:** Kinetic parameters of VLDL-apoB100 (fasted and fed states), TRL-apoB48 and TRL-apoA-I (fed state).

_Parameters_	_Fasted state_	_Fed state_
_VLDL-apoB100 FCR (pool/day)_	_6.94_ ± _1.19_	_7.72_ ± _1.68_
_VLDL-apoB100 pool (mg/kg)_	_76 .4_ ± _13_	_125.9_ ± _16_
_VLDL-apoB100 APR (mg/kg/day)_	_7.9_ ± _1.4_	_15.9_ ± _4.8_
_TRL-apoB48 APR (mg/kg/day)_	–	_1.54_ ± _0.26_
_TRL-apoA-I APR (mg/kg/day)_	–	_2.20_ ± _0.61_
_TRL-apoB48 FCR (pool/day)_	–	_6.70_ ± _0.93*_
_TRL-apoA-I FCR (pool/day)_	–	_4.29_ ± _1.09_
_Absolute apoA-I transfer TRL_ → _HDL (mg/kg/day)_	–	_1.40_ ± _0.48_

Data from six healthy subjects are shown as the mean ± SD. *p < 0.05 TRL-apoB48 FCR compared with TRL-apoA-I FCR.

**Table 2:** Kinetic parameters of HDL-apoA-I subclasses in fasted and fed states.

_Parameters_	_Fasted state_	_Fed state_
_Conversion preβ1_ → _α-HDL (pool/day)_	_85.40_ ± _12.68_	_66.56_ ± _14.72_
_Conversion α_ → _preβ1-HDL (pool/day)_	_4.46_ ± _1.48_	_4.70_ ± _0.91_
_Preβ1-HDL-apoA-I FCR (pool/day)_	_0.45_ ± _0.07_	_0.23_ ± _0.04*_
_α-HDL-apoA-I FCR (pool/day)_	_0.29_ ± _0.03_	_0.34_ ± _0.03_
_Preβ1-HDL-apoA-I pool size (mg/kg)_	_6.94_ ± _1.17_	_7.21_ ± _0.50_
_α-HDL-apoA-I pool size (mg/kg)_	_54.47_ ± _1.44_	_52.94_ ± _5.69_
_Hepatic apoA-I APR (mg/kg/day)_	_18.67_ ± _1.69_	_17.17_ ± _2.75_
_Intestinal_^a^_apoA-I APR (mg/kg/day)_	–	_2.20_ ± _0.61_
_Total apoA-I APR (mg/kg/day)_	_18.67_ ± _1.69_	_19.37_ ± _2.47_

Data from six healthy subjects are shown as mean ± SD for each state. *p < 0.05 between fasted and fed state.

HDL, high-density lipoprotein; FCR, fractional catabolic rate; APR, absolute production rate.

^a^Estimate of intestinal apoA-I production related to feeding. This value does not account for a basal secretion from the enterocytes in a fasted condition.

### Correlations

As expected, plasma TG significantly correlated with plasma apoC-III concentrations (r = 0.70, *p* = 0.019). As shown in Table 3, no significant correlation was observed between apoC-III plasma concentrations during the fed state and TRL-apoB48, TRL-apoA-I kinetics or intestinal apoA-I synthesis. No significant correlation was observed between any of the HDL-apoA-I kinetic parameters and apoC-III plasma concentrations for fasted and fed groups. Of note, HDL-apoC-III was significantly and negatively correlated with the conversion of α-HDL particles to preβ_1_-HDL (r = − 0.73, *p* = 0.019).

**Table 3:** Spearman correlations obtained between kinetic parameters and main plasma lipids.

Parameters	TC	TG	HDL-C	LDL-C	Plasma apoC-III	HDL-apoC-III
TRL-apoB48 FCR	ns	ns	0.93 (0.019)	ns	ns	ns
TRL-apoB48 APR	ns	ns	ns	ns	ns	ns
TRL-apoA-I FCR	ns	ns	ns	ns	ns	ns
TRL-apoA-I APR	ns	ns	ns	ns	ns	ns
Absolute apoA-I transfer TRL → HDL	ns	ns	ns	ns	ns	ns
Preβ_1_-HDL-apoA-I FCR	ns	ns	ns	ns	ns	ns
α-HDL-apoA-I FCR	ns	ns	ns	ns	ns	ns
Conversion preβ_1_ → α-HDL	ns	ns	ns	ns	ns	ns
Conversion α → preβ_1_-HDL	ns	ns	ns	ns	ns	− 0.73 (0.019)
Hepatic apoA-I APR	ns	ns	ns	ns	ns	ns
Intestinal apoA-I APR	ns	ns	ns	ns	ns	ns
Total apoA-I APR	ns	ns	ns	ns	ns	ns

TRL, triglyceride-rich lipoprotein; HDL, high-density lipoprotein; FCR, fractional catabolic rate; APR, absolute production rate; TC, total cholesterol; TG, triglyceride; HDL-C, HDL cholesterol; LDL-C, LDL cholesterol; ns, not significant.

Values are r coefficients (*p*-values).

## Discussion

In this randomized study, we investigated preβ_1_-HDL-apoA-I and α-HDL-apoA-I human kinetics in fasted and fed states and estimated the relative intestinal contribution of apoA-I total synthesis. Except for preβ_1_-HDL-apoA-I clearance rate, we observed similar kinetic data for HDL-apoA-I for both the fed and fasted states. We estimated a production rate of apoA-I from the intestine during the fed state to be around 13% of the overall production rate. Plasma apoC-III concentrations increased with feeding, but we did not observe any significant correlation between apoC-III concentrations and apoA-I kinetic data except for HDL-apoC-III that was inversely correlated with the conversion of α-HDL to preβ_1_-HDL. However, the low number of subjects enrolled here constitutes a major limitation of the study stemming primarily from the complexity of kinetic protocols.

We have adapted a model for studying apoA-I kinetics including preβ_1_-HDL^13^. Although unexpected, we had to include two pools of preβ_1_-HDL to get better fits probably because of some heterogeneity in these particles. This is a limitation of the FPLC method we have used for the sample preparation. For the fasted experiment, we assumed that the main source of apoA-I was the liver but we cannot exclude an intestinal production as suggested in animal models^14,15^. Then, we can only estimate the production related to the meal stimulation. As a precursor pool, we used the enrichment of the intracellular hepatic leucine estimated by the enrichment plateau of VLDL-apoB100^16^. For the fed state model, we added intestinal apoA-I synthesis to the hepatic synthesis. For the intestinal precursor pool, we used the enrichment plateau of TRL-apoB48. TRL-apoB48 kinetic parameters were similar to those previously reported^17–19^, validating our methods for measuring apoB48 enrichments. In this model, we assumed that intestinal apoA-I was incorporated directly into TRL particles because a direct synthesis of intestinal HDL-apoA-I could not be identified, regardless of the various models we have tested. This could represent another limitation of our study. Along the same lines, while an exchange of apoA-I between the preβ_1_-HDL, α-HDL, and TRL pools was necessary to obtain a better fit of the enrichment curves, estimates of the transfer rates of apoA-I between HDL and TRL were difficult to obtain, and large standard deviations were observed. This is another potential limitation of the model. However, for all subjects, there was an absolute net transfer of apoA-I from TRL to HDL.

We did not observe any major differences in plasma apoA-I concentrations or apoA-I kinetics between the fed and fasted states in line with a previous report^20^. Cohn et al., with a similar study design, did not observe any change in total HDL-apoA-I kinetics in the fasted and fed states^3^. However, their model used the enrichment plateau of VLDL-apoB100 as a forcing function for the precursor pool, assuming that the single source of apoA-I was the liver. Although this might be true in the fasted state, the intestinal production of apoA-I is probably significant in the fed state. Using a more complex model that includes apoA-I production from the liver and the intestine, we reached the same conclusion. It can be concluded their simple model is relevant for the overall HDL-apoA-I catabolic or synthesis rates. Of note, similar ranges for data were already previously reported in fasted^21–24^ and fed^25,26^ kinetic studies, but with no direct comparison between fasting and feeding. In addition, we added information on preβ_1_-HDL-apoA-I kinetics, which revealed a decrease in the FCR with feeding, but with no related change in α-HDL mass or synthesis.

The metabolism of the preβ_1_-HDL-apoA-I is complex as they can be excreted through the kidney or recycled into the plasma (i.e. interconversion of HDL particles in the plasma compartment) via hepatic lipase, cholesterol ester transfer protein, phospholipid transfer protein, and phospholipids or HDL-apoM^27^. Most of these factors could be modulated through diet and their specific roles deserve further investigations.

To the best of our knowledge, three studies have looked at the kinetic metabolism of preβ_1_-HDL-apoA-I and mature HDL with an endogenous labeling of apoA-I with a stable isotope tracer. Two studies have used a constant infusion of the tracer and blood sample collection over 10 h to 14 h in fasted subjects^13,28^ while a large bolus injection of the tracer over 10 min was used in the last one associated with blood sampling over 96 h in non-fasted patients^29^. Similar kinetic data were observed in the first two studies but controversial results were reported in the third one. This could be attributed to the different study designs and blood sample collection or a larger number of analyzed HDL particles but the modeling is probably the main reason of this discrepancy. Although a clear relation between precursor and product was reported in monkeys between preβ_1_-HDL-apoA-I and α-HDL-apoA-I with exogenous labeled native HDL with radiotracers^30^, the authors challenged the canonical stepwise enlargement and contraction model for HDL metabolism. From the similarity of the tracer enrichment curves in the different particles, as we have also observed in this study, they suggested a direct and large entry of apoA-I into the mature HDL. Their conclusion can be challenged because the subjects were not fasted and they did not take into account any exchanges with the TRL-apoA-I. In the different models they have tested, in one of them close to our approach, a good fit was also observed. Nevertheless, they decided to discard this model because of its simplicity. Finally, they assumed that the similarity of the tracer enrichment curves means a direct entry in each particle. This could be also related to very fast exchanges and equilibrium between the circulating particles while the labeled protein is slowly synthesized as apoA-I. A recent in vitro study has studied the conversion rate of nascent to mature HDL in BHK-ABCA1 cells^29^. The conversion was solely to mature HDL and very fast (t_½_ < 10 min) and in a range similar of the preβ_1_-HDL-apoA-I residence time we have observed. Beside these discrepancies, these results altogether raised a limit of the endogenous labeling of slow synthesis proteins incorporated in circulating carriers with fast exchanges and turnover rates. New study designs and modeling should be probably proposed in this particular situation.

Our model suggests the increased intestinal apoA-I production related to meal stimulation must be first incorporated into TRL particles and then exchanged with mature HDL. This assumption may represent a limitation of our model. Studies in animal models have observed a synthesis of apoA-I and HDL within the enterocytes accounting for 30% of the circulating pool^14^ but evidences supporting a direct synthesis of HDL particles from the intestine in humans are not strong. Preβ_1_-HDL and α-HDL have been identified in the peripheral lymph^31^. However, due to a filtration process, the peripheral lymph HDL profile seems to reflect the plasma HDL profile and, in contrast to the rat model, nascent HDL particles were not observed in human samples of thoracic duct lymph^32,33^.

We estimated that intestinal apoA-I synthesis, shuttling through TRL particles, accounts for approximately 13% of the total apoA-I production with a meal stimulation. Green et al*.* estimated that about 50% of apoA-I originated from the intestine in chyluric patients, but this is a disease-state model^34^. Anderson et al*.* assumed that only apoA-I in association with lymph chylomicrons represents the contribution of intestinal synthesis, and was estimated to be around 26% in patients with thoracic duct drainage^32^. Again, this result could be challenged because of the apoA-I filtration process from plasma to lymph. Consistent with our data, Ikewaki et al*.* estimated that the intestine contributed to 10% of the total apoA-I pool by using dual stable isotope and radiotracer infusion^35^. However, when Velez-Carrasco et al*.*^25^ performed a single tracer infusion study of apoA-I kinetics within TRL and mature HDL (but not preβ_1_-HDL) in a fed state, they found no exchange of apoA-I between HDL and TRL. Furthermore, they did not identify a direct catabolism of apoA-I from TRL or a direct production of mature HDL from the intestine. Although their measured rates of total apoA-I synthesis and catabolism were similar to our data, they estimated that intestinal apoA-I synthesis reflects only 0.15% of the total synthesis of apoA-I. This large discrepancy could be explained by differences in the compartmental analysis models, experimental protocol lengths, and experimental diets or, as acknowledged by the authors, some very fast exchanges not able to be captured with the experimental protocol. An accurate measurement of intestinal apoA-I synthesis would have required a specific labeling within the intestine. However, specific tracers of protein synthesis targeting only this tissue have not yet been identified.

The increase in plasma apoC-III concentrations upon feeding was previously reported and likely reflects an increase in apoC-III synthesis^10,11^. ApoC-III is an inhibitor of lipoprotein and hepatic lipases and increases the assembly and production of VLDL from the liver^36,37^. Overall, apoC-III increases plasma TG levels^9^. Previous kinetic studies reported a significant inverse correlation between HDL-apoA-I catabolic rates and HDL-apoC-III concentrations^38^. In line with this report, we found an inverse correlation between HDL-apoC-III and the conversion of α-HDL to preβ_1_-HDL particles. However, whether this finding is related to a direct effect of apoC-III on apoA-I metabolism, or an indirect effect due to changes in the TG content of HDL or plasma remains unknown. Recent studies with an antisense oligonucleotide showed an increase in HDL-C associated with a sharp reduction in plasma apoC-III^12^. Therefore, decreases in plasma apoA-I and HDL-C levels could be expected with the increase in apoC-III levels observed with feeding but we did not observe this lipid profile during the feeding experiment. Furthermore, no significant correlation was found between plasma apoC-III concentration and catabolic rates of apoA-I in mature or preβ_1_-HDL. The limited absolute increase in plasma apoC-III concentrations and the small number of subjects in this study could account for this lack of association. Kinetic studies with apoC-III inhibitors would be more appropriate to address this question.

In conclusion, we did not observed any major differences in global apoA-I kinetic parameters (synthesis and catabolism) between the fed and fasted states. This observation validates the comparison of kinetic data concerning apoA-I obtained from different studies, regardless of differences in their experimental protocols and food intake. Until a method of direct and specific labeling of intestinal apoA-I will be established, our approach and model, albeit some limitations, provide a reasonable estimate of intestinal apoA-I synthesis stimulated by a meal. Finally, this study raised some limitations for the kinetic study of very fast turnover lipoproteins with an endogenous tracer labeling.

## Materials and methods

### Patients

Six healthy male subjects (age: 26.3 ± 5.7 years, body mass index: 21.1 ± 2.5 kg/m^2^) with normal plasma lipid concentrations (TC: 164 ± 30 mg/dL, TG: 75 ± 27 mg/dL, HDL-C: 53 ± 10 mg/dL, low-density-lipoprotein cholesterol (LDL-C): 93 ± 32 mg/dL, apoA-I: 143 ± 13 mg/dL, and apoB100: 69 ± 20 mg/dL) and without medication were included in a crossover design study. They were submitted to two explorations within 2 weeks described later as the fasted and feeding protocols. The order of the first exploration (fasted or feeding) was randomly assigned. The experimental protocol was approved by the ethics committee of Nantes University Hospital. A written consent was obtained from each volunteer before inclusion in the study (CPPRB Pays de Loire, reference 0266). All methods were carried out in accordance with guidelines and regulations of the ethics committee of Nantes University Hospital.

### Experimental design

After an overnight fasting (12 h), subjects received a primed bolus of ^2^H_3_-leucine at 10 µmol/kg (99.8 atom %; Cambridge Isotope, MA, USA), immediately followed by a constant infusion at 10 µmol/kg/h for 14 h. For the feeding protocol, a 2,200-kcal meal composed of Slim Fast, cheese, and toasted bread was divided into 16 equal portions. Each meal portion was carefully weighed and contained 44% carbohydrate, 15% protein, and 41% fat by calories corresponding to 1/18 of the total calorie intake. After the 12 h overnight fasting, subjects received a meal portion at 2 h and 1 h before bolus tracer injection (t = 0 h). Then, one meal portion was given every hour during tracer injection (t = 0 h to 13 h). Venous blood samples (5 mL) were drawn into EDTA tubes (Venoject, Paris, France) at baseline (t = 0 h), every 15 min during the first hour, every 30 min during the next 2 h, and then hourly until the completion of the study. All blood samples were withdrawn before food intakes. The infusion protocol was identical for the fasted state experiment, but no meal portions were given (subjects were given free access to water). Over the next 4 days, fasted and fed subjects resumed their normal diets, and a daily blood sample was collected (t = 24 h, 48 h, 72 h, and 96 h).

### Sample preparation and storage

Immediately after blood collection, plasma was separated by centrifugation for 30 min at 4 °C. Sodium azide, an inhibitor of bacterial growth, and a protease inhibitor were added to plasma samples at final concentrations of 1.5 and 0.5 mM, respectively. All samples were stored at − 80 °C until use.

### ApoC-III measurements

ApoC-III was quantified by trypsin proteolysis and liquid chromatography-tandem mass spectrometry analysis of signature peptides as previously described and validated^39^.

### Isolation of HDL subclasses

HDL subclass separation was performed by fast protein liquid chromatography (FPLC) on a Superdex 200 h 10/30 column (Amersham Pharmacia Biotech Inc., Orsay, France). Elution buffer contained 1 mM EDTA, 154 mM NaCl, and 0.02% NaN_3_ and was pre-filtered through a 0.22-µm GV membrane filter (Duropore, Millipore, Billerica, MD, USA). Plasma samples (200 µL) were injected, eluted at a flow rate of 0.35 mL/min, and 0.2-mL fractions were collected. ApoA-I contents in FPLC fractions were measured by immunoturbidimetry. Details on FPLC profiles and the purity of collected fractions have been described previously^40^. To only analyze pure HDL subclasses, 10 fractions, assumed to be a mixture of α-HDL and preβ_1_-HDL, were not considered. With this exclusion criterion, 52% of total plasma apoA-I was recovered, with 89% of apoA-I in α-HDL and 11% in preβ_1_-HDL. To determine the concentrations of apoA-I within preβ_1_- and α-HDL subpopulations, we multiplied these percentages (89% for α-HDL and 11% for preβ_1_-HDL) by the total plasma concentration of apoA-I. To confirm the purity of the subclasses, non-denaturing two-dimensional polyacrylamide gel electrophoresis (PAGE) was performed on α-HDL and preβ_1_-HDL FPLC fractions. After electrophoresis, lipoproteins were transferred onto a nitrocellulose membrane. An anti-apoA-I antibody was applied to confirm that preβ_1_-HDL particles were not found in the α-HDL sample and reciprocally, as previously described^40^.

### Isolation of TRL by ultracentrifugation

The density of plasma was adjusted to 1.006 g/mL with a NaCl solution. Very-low-density lipoproteins (VLDL) were isolated by ultracentrifugation using a LKB ultracentrifuge with a RP55T rotor at 40,000 rpm for 24 h at 4 °C. For plasma from the fed group, this fraction contained chylomicrons and, therefore, was referred to as the TRL fraction. VLDL-apoB100 concentrations were measured by immunoturbidimetry and TRL-apoB48 concentrations were determined by gas chromatography-mass spectrometry (GC–MS) as previously described^41^.

### Isolation and preparation of apolipoproteins

ApoA-I was resolved from the purified HDL subclass preparations by a 4–20% gradient sodium dodecylsulfate (SDS)-PAGE. ApoB100 and apoB48 were resolved from the VLDL and TRL preparations using a 4–10% gradient SDS-PAGE. These apolipoproteins were first identified by immunoblotting with an antibody against human apoB48/B100 (Biodesign, Saco, ME). In parallel, apolipoproteins were also isolated by SDS-PAGE and stained with Coomassie Brilliant Blue R 250 (Sigma Aldrich, Saint-Quentin Fallavier, France). Apolipoproteins were identified by comparing migration distances with known molecular weight standards (electrophoresis calibration kit, Pharmacia LKB, Biotechnology Inc., Piscataway, NJ, USA). The apoA-I, apoB100, and apoB48 bands were then excised from the polyacrylamide gels and hydrolyzed for 24 h in 4 N HCl at 110°C^16,23^. Hydrolysates were used to determine tracer-to-tracee ratios.

### Measurements of tracer enrichments

Hydrolysates were dried under nitrogen and amino acids were purified by cation exchange chromatography using a Dowex 50WX8-200 resin (Sigma Aldrich). Amino acids were esterified with propanol/acetyl chloride and derivatized using heptafluorobutyric anhydride (HFBA, Fluka, Saint-Quentin Fallavier, France). GC–MS enrichment measurements were performed on a 5890A gas chromatograph connected with a 5971A quadrupole mass spectrometer (Hewlett-Packard, Palo Alto, CA). The isotopic ratio was determined by the selected ion-monitoring mode at mass-to-charge ratios of 282 and 285 for endogenous and labeled leucine, respectively^13,40^.

### Model assumptions and kinetic analysis

**Figure 5:**
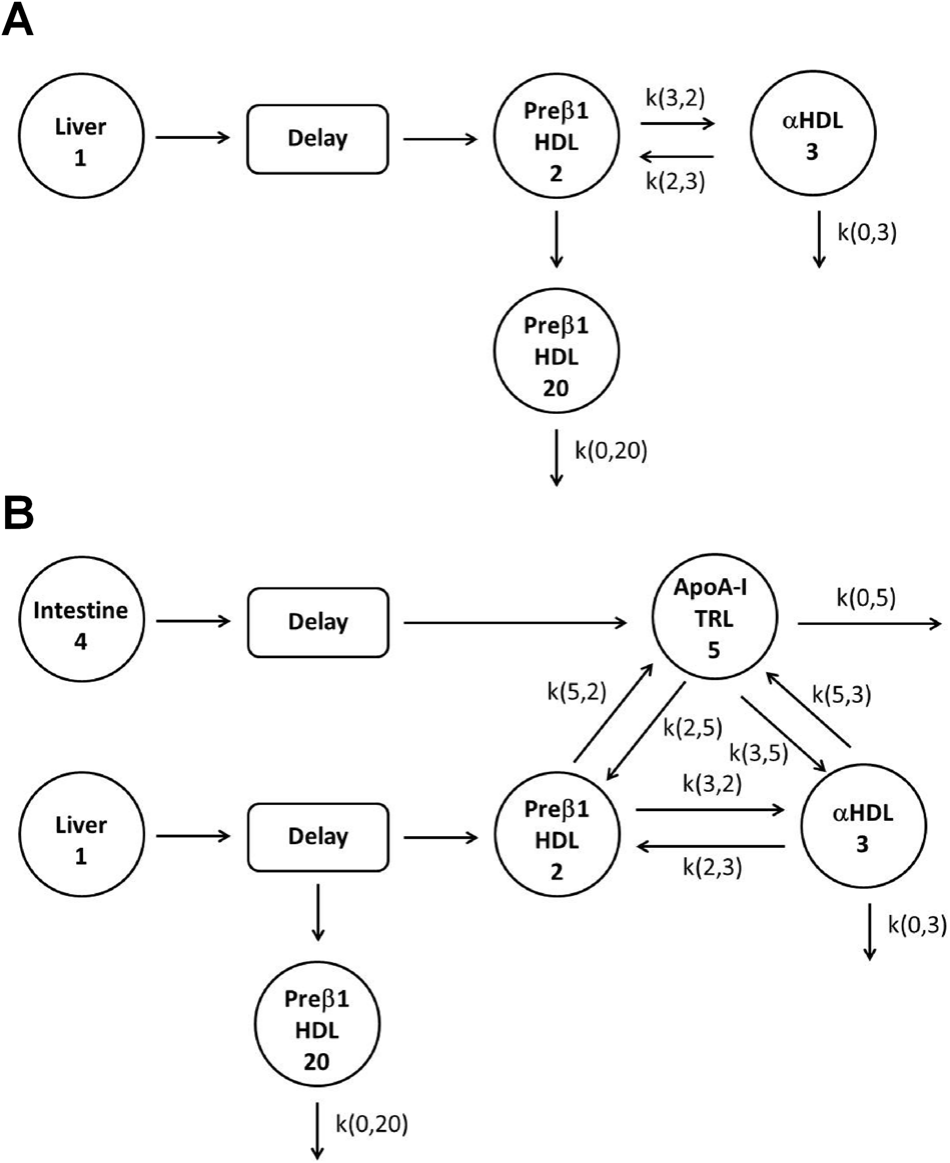
Multi-compartmental model used to analyze kinetics of apolipoprotein A-I (apoA-I) in the fasting (**A**) and the fed (**B**) states.

Kinetic analysis of tracer-to-tracee ratios was achieved by using computer software for simulation, analysis, and modeling (SAAM II, version 1.0.1, Resource Facility for Kinetic Analysis, Department of Bioengineering, SAAM Institute, Seattle, WA, USA). Different models were tested and we selected two of them for the fasted and fed states giving the best goodness-of-fit measures according to Akaike Information Criterion. The multi-compartmental model used for studying apoA-I metabolism is shown in Fig. 5A for the fasted experiment^13^. This model was adapted (Fig. 5B) for the fed experiment. Compartments 1 and 4 represent apolipoprotein precursor pools defined respectively by a forcing function representing asymptotic enrichment plateau from either VLDL-apoB100, that only originates from the liver (fasted and fed experiments), or TRL-apoB48, the truncated form of apoB100 that only originates from the intestine (fed experiment only). Intracellular delay compartments represent the time required for apoA-I synthesis and secretion from the liver and intestine. Compartments 2 and 3 are tracer-to-tracee enrichment curves of apoA-I within the preβ_1_-HDL and α-HDL populations, respectively. The model allowed reversible transfer of apoA-I between HDL subclasses. Presumption of heterogeneity of preβ_1_-HDL generated a better fit to the data, but did not dramatically affect the kinetic parameters. Then, pools 2 and 20 were associated with the apoA-I–preβ_1_-HDL tracer-to-tracee ratio. Their pool sizes were calculated by the model assuming their sums were equal to the total apoA-I–preβ_1_-HDL pool. In the fed experiment model (Fig. 5B), compartment 5 represents TRL-associated apoA-I (tracer-to-tracee ratio and mass). Exchange of apoA-I between the TRL and HDL compartments and direct catabolism of apoA-I from the TRL and HDL compartments were included in the model to obtain a better fit. The apoB48 kinetics were obtained from a simple model with two compartments. Tracer-to-tracee ratios of VLDL-apoB100 were fitted to a mono-exponential function, defined as A(t) = Ap(1 − e^[-k(t-d)]^), where A(t) is the apolipoprotein enrichment at time t, Ap is the enrichment of the tissue precursor amino acid pool from which the protein was derived (estimated from VLDL-associated apoB100 enrichment curve at plateau), d is the delay between the beginning of the experiment and the appearance of tracer in apolipoprotein, and k is the fractional synthetic rate (FSR) of apolipoprotein. The fractional production rate (FPR), defined as the proportion of apoA-I entering the pool per unit time (pool/day), and the absolute production rate (APR), defined as the amount of apoA-I entering the pool per unit time (mg/kg/day), were estimated. APR is the product of the FPR and the pool size of apoA-I in each HDL subclass. The apoA-I pool size (mg/kg) was calculated by multiplying the mean HDL subclass apoA-I concentration by 0.045 (L/kg), based on an assumed plasma volume of 4.5% of body weight^42^. The apoA-I pool size was considered to be constant, as no significant variation was observed between measurements made at different sampling times in the fasted and fed states. Under these steady-state conditions, FPR equals the fractional catabolic rate (FCR). Rates for apoA-I clearance and recycling within the HDL subclasses or apoA-I exchange between HDL and TRL were estimated and expressed in units of pool/day. The clearance rate reflects the direct removal of apoA-I [k(0,20); k(0,3); k(0,5) in Fig. 5]. The residence time (RT) was 1/FCR. ApoA-I recycling corresponded to conversion between preβ_1_-HDL and α-HDL [k(3,2); k(2,3)]. ApoA-I exchange between HDL and TRL was characterized by four parameters [k(2,5); k(5,2); k(3,5); k(5,3)], but was expressed as the absolute apoA-I transfer rate between TRL and HDL (mg/kg/day).

### Statistical analysis

Data are reported as the mean ± standard deviation (SD), unless otherwise specified. The Wilcoxon matched-pairs signed rank test (significance at p < 0.05) was used to determine differences between groups using GraphPad Prism software (version 6.0, GraphPad Software Inc., La Jolla, CA, USA). Correlations were evaluated by the Spearman test.

## Data Availability

The datasets analyzed during the current study are available from the corresponding author on reasonable request.
